# Synthesis and Performance of Iron Oxide-Coated Ceramsite in a Biotrickling Filter for Nitric Oxide Removal under Thermophilic Conditions

**DOI:** 10.3390/ma11030359

**Published:** 2018-02-28

**Authors:** Han Li, Ze Guo, Dafu Wu, Jing Fan, Shaobin Huang, Shaofeng Zhou

**Affiliations:** 1 Postdoctoral Research Base, Henan Institute of Science and Technology, Xinxiang 453003, China; lihanenjoy@hist.edu.cn; 2 School of Environment, Henan Normal University, Xinxiang 453007, China; fanjing@henannu.edu.cn; 3 Department of Human Resources, Henan Institute of Technology, Xinxiang 453003, China; h34@scut.edu.cn; 4 School of Environment and Energy, South China University of Technology, Guangzhou 510006, China; chshuang@scut.edu.cn (S.H.); wangfei@hist.edu.cn (S.Z.)

**Keywords:** iron oxide-coated porous ceramsite, biofilter, microbial growth, NO removal

## Abstract

A novel medium consisting of iron oxide-coated porous ceramsite (modified ceramsite) was investigated for NO removal under thermophilic conditions in this study. We used a surface coating method with FeCl_3_·6H_2_O as the modifier. When ceramsite was calcined for 4 h at 500 °C, the surface pH value decreased to 3.46, which is much lower than the isoelectric point of ceramsite, ensuring its surface was electropositive. The surface of modified ceramsite changed from two- to three-dimensional and exhibited excellent adsorption behavior to assist microbial growth; the maximum dry weight of the biofilm was 1.28 mg/g. It only took 8 days for the biofilter constructed from the modified ceramsite to start up, whereas that packed with commercial ceramsite took 22 days. The NO removal efficiency of the biofilter did not decrease apparently at high NO inlet concentration of above 1600 mg/m^3^ and maintained an average value of above 90% during the whole operation period. Additionally, the morphological observation showed that the loss of the surface coating was not obvious, and the coating properties remained stable during long-term operation. The maximum NO inlet loading of the biotrickling filter was 80 g/(m^3^·h) with an average removal efficiency of 91.1% along with a quick start-up when using the modified ceramsite filler. Thus, modified ceramsite can be considered a very effective medium in biotrickling filters for NO removal.

## 1. Introduction

With the rapid industrial development achieved in recent years, the emission of flue gas has increased, which is strongly linked to air pollution levels. Flue gas from a powerplant consists of CO_2_, SO_x_, NO_x_ and O_2_, and the primary pollutants are SO_x_ and NO_x_. NO is the primary composition of NO_x_, particularly to flue gas, and accounts for about 90% [1]. Nitrogen oxides (NO_x_) from flue gas can cause acid rain and are the main substance during the formation process of photochemical smog. Besides, NO_x_ can destroy the ozone, which brings a threat to human health and the environment [2]. For a long period of time, chemical and physical methods are the basis for the purification treatment of NO_x_ [3]. The typic techniques of simultaneous desulfurization and denitrification are widely used in atmospheric control [4], while being prohibitively expensive when dealing with large volumes of flue gas [5]. Furthermore, secondary pollutants can be produced and often need further treatment [6]. There have been emerging various new technologies to reduce NO_x_ from flue gas [7,8,9,10,11]. Advances in microbiology, especially associated with the isolation of aerobic denitrifying bacteria, have revealed that microbial purification of volatile pollutants is a promising technique [12,13]. Microbial denitrification has rapidly become a new research focus. Bioprocesses using biotrickling filters and biofilters are emerging post-combustion control technologies that are potential alternatives for purification of gases containing small amounts of NO_x_ [14,15].

The packing material is the carrier of microorganisms in biofilters, and is the main location of immobilization and pollutant treatment [16]. Therefore, it is of great significance to choose suitable packing materials for effective operation of biofilters. To select a good packing material should consider the following factors: (a) packing material type, (b) packing porosity, (c) packing moisture capacity, (d) packing nutrient content, and (e) sorption characteristics of the packing surfaces. In addition, besides, it is essential to study the adsorption characteristics of the target chemical by the packing material to determine a suitable packing material.

A modified packing material is a microbe carrier surface coated with a modifier by physical and chemical reactions, thereby changing the primary physical and chemical properties of the packing material particle surface. Such modification can enhance the immobilization ability of the material and its adsorption capacity for certain specific substances. These favorable changes mean that packing material modification is widely used in wastewater treatment processes. Research has shown that Fe_2_O_3_ is more sensitive to bacteria than other packing material [17], which is probably because of its relatively higher point of zero charge (PZC) [18]. When an electrolyte oxide or amphoteric species is placed in water, the zwitterionic electric charge could change because of the different pH value of the solution. In addition, an electric double layer can form on the surface and generate electrophoresis when a voltage is applied. Adding some electrolyte to the water will compress the counter ion layer of the electric double layer. Conversely, adding a certain number of ions will result in minimum compression of the counter ion layer, a surface potential of zero, and no electrophoresis; this status is referred to as PZC. At this time, the solution pH is the pH of the isoelectric point of the oxide, which is indicated as PI. When the solution pH is higher than the PI (pH > PI), amphoteric ions release protons and are negatively charged. When the pH of the solution is lower than the PI (pH < PI), zwitterions are protonated and thus positively charged. It was found that cell-induced and supernatant-induced reductions were combined to achieve favorable results in a biofilter modified with Fe_2_O_3_ [17]. Therefore, the use of Fe_2_O_3_ as a modifier to improve the surface properties of biofilters is attracting increasing attention [19]. In addition, Chen [20] assessed the effectiveness of sand covered with aluminum hydroxide by in situ precipitation over four months, and during this period, the sand was exposed to wastewater. Biogrowth in one set of columns was prevented by receiving chloride wastewater, while a parallel column was used to treat dechlorination wastewater. The results showed that the aluminum content of coated sand decreased by about 25% in the first two weeks, and remained relatively stable, much higher than that of uncoated sand. Similarly, the zeta potential of the coated sand decreased from above +20 mV to below −70 mV in the first two weeks, which was still much more electropositive than that of uncoated sand. In the absence of biogrowth, the zeta potential of coated sand subsequently remained approximately unchanged, and most importantly, it exhibited excellent performance for bacteria removal. This study assesses the technological potential and economic implications of metallic hydroxide coating of filter media.

The natural inert packing material typically used in biotrickling filters include rock wool-compost [21], zeolite [22], and the compound packing material lightweight ceramsite [23]; most of these are silicate minerals or organic polymer and are negatively charged. Generally, it is believed that the adsorption capacity of such packing material for negatively charged bacteria is weak; after modification, their adsorption capacity can be greatly increased. Modification technology has gradually been developed for nearly a decade. The basic theory of such modification is to change the physical and chemical properties of the original packing material or its surface by physical and chemical methods [24,25]. Guzek [26] analyzed modified packing material and noted that the physical and chemical properties of the surface coating affected filler adsorption capacity. Because the coating had a large surface area, numerous surface adsorption sites, and high surface roughness and porosity, the modified packing material exhibited great advantages over their unmodified equivalents in biofiltration. Modified fillers can be divided into two categories: one is the addition of components during the manufacturing process, such as nano-modified ceramics and modified polypropylene biological packing material. The other involves changing the surface properties of the filler matrix. For example, activation of modified zeolite membranes at 500 °C was found to effectively promote catalytic cracking deposition of silane in the zeolitic pores, which resulted in considerable improvement of adsorptive performance [27]. There have been multiple investigations of modified packing material used for wastewater treatment; however, their application in gas pollutant treatment has seldom been reported [28,29,30].

**Figure 1:**
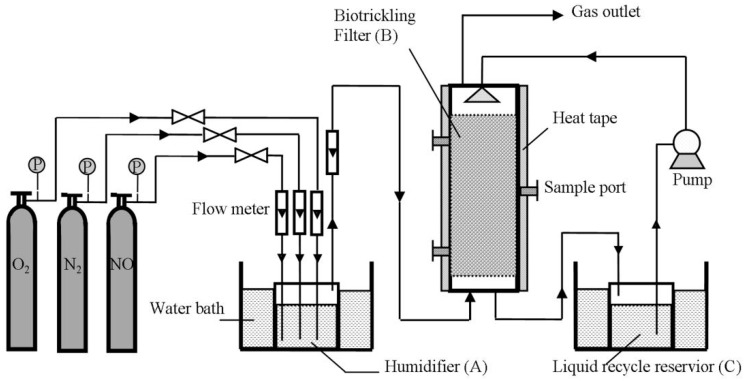
Experimental setup of a biotrickling filter system.

Packing material modification is beneficial to improve packing physical parameters, enhance the biofilm capacity in a reactor, and effectively increase the reaction area of pollutants and biological membranes. Thus, packing material modification affects biological filter processing capacity, buffering capacity, and the volume load of the whole biofilter system, ultimately improving the removal ability of NO_x_ by a biological trickling filter. In this study, we selected a commonly used commercial ceramic as the matrix for modification, as it has a regular shape and stable property, most of all, it could be easily achieved with low lost. We investigate the surface modification method to improve the surface properties of the packing material to provide a more favorable growth environment for microorganisms, and then use the modified material in a thermophilic biofilter reactor, as shown in Figure 1. This novel biofilter is used to treat NO from simulated flue gas under thermophilic conditions. The performance of the biofilter, including the start-up rate and NO removal efficiency, is investigated. The coating properties after a long period of operation are examined. The results of this study provide theoretical guidance for the practical application of iron oxide-based ceramsite in biotrickling filters for NO removal.

## 2. Materials and Methods

### 2.1. Microbes

The strain *Chelatococcus daeguensis* TAD1 was isolated by our group from the biofilm of an on-site biotrickling filter at a coal-fired power plant (Guangzhou, China). The 16S rRNA sequences (1385 bp) of *C. daeguensis* were searched for similarities in blastn (NCBI, Bethesda, MD, USA). The sequence data for the strain has been submitted to the DDBJ/EMBL/Gen-Bank databases under accession No. HM000004 [31].

### 2.2. Growth Medium

The trickling nutrient liquid included the following components (in g/L): KNO_3_, 1.0 (in the start-up stage); NaCl, 4.7; disodium succinate, 5.0; Na_2_HPO_4_, 7.9; KH_2_PO_4_, 1.5; MgSO_4_·7H_2_O, 0.1; 1 mL of trace element solution. The trace element solution consisted of (in g/L): EDTA, 50.0; CaCl_2_, 5.5; MnCl_2_·4H_2_O, 5.06; FeSO_4_·7H_2_O, 5.0; ZnSO_4_, 2.2; CoCl_2_·6H_2_O, 1.61; CuSO_4_·5H_2_O, 1.57; (NH_4_)_6_Mo_7_O_2_·4H_2_O, 1.1. All chemicals were analytical-grade reagents, commercially available, and used without further purification. NO (99.9%) was obtained from Foshu Kede Gas Co., Guangzhou, China. N_2_ (99.99%) and O_2_ (99.99%) were obtained from Guangzhou Gas Co., Guangzhou, China.

### 2.3. Biotrickling Filter Setup and Operation

The bench-scale biofilter reactor shown in Figure 1 was used in this study. The reactor was constructed of cylindrical plexiglass. The height and diameter of the reactor were 80 and 10 cm, respectively. Iron oxide-coated ceramsite (or commercial ceramsite as a reference) was used as the packing material. The packing space was in the height of 20 cm. The reactor was wrapped with heating tape, and then covered with a layer of fiberglass as insulator. The heating tape was controlled by a digital temperature controller, which maintained the reactor temperature at 50 ± 1 °C.

Inlet gas was obtained by mixing pure NO, N_2_, and O_2_, while the three gases were mixed in a humidifier before entering the reactor. The simulated inlet gas was preheated by passing through the humidifier, which was set in a thermostatic water bath (50 ± 1 °C). The inlet NO concentration was controlled by adjusting the flow rate of each gas through the flow meter before entering the humidifier. The constant-volume (3 L) liquid reservoir for nutrient recycling was also controlled at 50 ± 1 °C by a thermostatic water bath. The recycling liquid was pumped to the upper surface of the packing materials by a peristaltic pump which was set at a flow rate of 250 mL/min to maintain sufficient moisture. A 500-mL aliquot of the liquid medium was drawn off and renewed with fresh nutrient solution containing 1% *C. daeguensis* TAD1 daily to confirm that the denitrifying strain *C. daeguensis* TAD1 was the dominant type of bacteria in the microbial community and provide necessary nutrients for microbial growth. The NO concentration in the influent gas was varied by regulating the mass flow meters. The main conditions for biotrickling filter setup and operation are listed in the Table 1.

### 2.4. Analytical Methods

Nitrate was analyzed by ion chromatography (DX-500; Dionex Corporation, Sunnyvale, UK). The column used was Dionex ionpac AS14. To measure the thermophilic removal of NO in a continuous gas stream, the NO, N_2_ and O_2_ fluxes were controlled by a mass flow controller (FM310-MT, Opine, Tianjin, China). The NO concentration at both the inlet and outlet was analyzed by a flue gas analyzer (350Pro, Testo, Baden-wurttemberg, Germany) at 10-h intervals. The NO concentrations at the inlet and outlet of the biofilter were also measured. The performance of the biofilter was quantified by its NO removal efficiency (RE) and Inlet loading, which were calculated as follows:

(1) $$\mathrm{RE}=\frac{C_{\mathrm{in}}-C_{\mathrm{out}}}{C_{\mathrm{in}}}\times 100\%$$

Inlet loading (L, g/(m^3^·h)):

(2) $$L=\frac{Q\cdot C_{\mathrm{in}}}{V}$$

where V is the packing volume of the filler in the biofilter reactor (L); Q is the gas flow rate (L/h), it was set at 60 L/h in this study; and C_in_ and C_out_ are the inlet and outlet NO concentrations (g/m^3^), respectively.

The biomass concentration attached to the packing materials was measured by the oven-drying method as follows [32]. Several ceramsites were removed from the biofilter to place in an oven at 105 °C, and then dried to a constant weight. W1 represented the dry weight, including the biofilm and carriers. The dry carriers were soaked in hydrochloride acid (1 mol/L) solution for 2 h at 80 °C, carried out ultrasonic cleaning for 1 h, and then rinsed with water until all the biofilm was cleaned away. Finally, drying treatment to the clean carriers until a constant-weight carrier was formed. The dry weight of the clean carriers was marked as W2. Thus, the biomass weight was obtained by subtracting W2 from W1. The biomass concentration attached to the packing materials was calculated as the ratio of the biomass weight to that of the dry carriers.

### 2.5. Modified Ceramic

#### 2.5.1. Ceramic Modification

The purpose of this experiment is to make the surface of ceramic with positive charge group composition. Therefore, the modified agent containing metal cations can be used. FeCl_3_·6H_2_O is cost low, and with low microbial toxicity, so we chose it as modifier. Ceramic modification involved three steps: surface pretreatment, soaking, and high-temperature calcination [28]. In the first step, the material was soaked in H_2_SO_4_ with a 1:500 volume ratio for 24 h, washed with water to a pH near neutral, and placed in a tray for drying at 105 °C. In the soaking step, the material was soaked with modifier of FeCl_3_·6H_2_O (1 mol/L) and then dried at a comparatively low temperature (usually 105 °C). The main purpose of this step is to induce the modifier to fully attach to the surface of the carrier. The material was stirred every 30 min to ensure full contact between the modifier and material. The surface preparation of selected carriers was conducted to restore the surface activity to achieve the strongest adhesion of the modifier. High-temperature calcination was performed to make the FeCl_3_ coating evaporate from the solution and then metal oxide was deposited on the filler surface. The calcination temperature and time directly affected the performance of the modified material.

#### 2.5.2. Evaluation Parameters of the Modified Ceramic

The preparation and use of modified ceramic require some of the physical and chemical parameters to be evaluated. Physicochemical parameters of fillers include porosity, density, surface pH, surface composition, isoelectric point (PI), and the amount of coating.

##### Surface pH

To measure the surface pH of the ceramic, 10 g of the selected ceramic was weighed accurately and placed in a beaker. Distilled water (90 mL) was added and then the suspension was stirred for 10 min. The pH of the solution was then measured, which was defined as the surface pH.

##### Porosity

Ceramic porosity was determined as the ratio between the pore volume and total volume of the ceramic. The filter layer gap ratio and filter particle shape, uniformity, and degree of compaction affect ceramic porosity. The measurement method is as follows:

First, measure the density of the ceramic: 100 mL (V1) water was added to the 200 mL-pycnometer, then put into 100 g dried ceramic slowly, tilt and shake the bottle to get rid of gas, read the bottle surface V2 after standing 24 h, which can have the formula of packing density ρ

ρ (g/cm^3^) = G/(V2 − V1) (3)

where G is mass of dried ceramic (g), V1 is the volume of added water (cm^3^), V2 is the volume after 24 h (cm^3^).

Once you have measured the density of the ceramic, put the ceramic after drying in the cartridge and the filler is filtered with water for some time, then measure the filter layer volume V. The porosity (m_0_) is calculated as follows

m_0_ (%) = 100 × (1 − G/ρV) (4)

where ρ is packing density of dried ceramic (g/cm^3^), V is the volume of filter layer volume (cm^3^).

##### Isoelectric Point

PI values were obtained by the analysis of empirical constants and potentiometric titration depending on the nature of the oxide.

##### Coating Content

Coating content (M) was determined per unit mass in units of mg/g. Coating amount directly affects the surface properties of the modified ceramic. To measure M, 50 g of ceramic was weighed accurately with an analytical balance, which is referred to as M1. M was calculated by the following equation,

M (mg/g) =1000(M2 − M1)/M1,(5)

where M1 is the ceramic mass before modification (g) and M2 is the ceramic mass after modification (g).

## 3. Results and Discussion

Usually, modification effect is evaluated according to the biomass amount when biofilm formation is completed. Besides, the experimental parameters for modified material before and after use including physical and chemical indicator nature, film forming properties, and pollutant removal can be compared.

### 3.1. Modification Conditions

The biotrickle reactor used for evaluating modification conditions is a smaller one with an operation period of 20 days. The height and diameter of the reactor were 50 and 8 cm, respectively. The packing space was in the height of 10 cm and the operation steps are just the same as the start-up period described in Section 2.3**.**

#### 3.1.1. Calcination Temperature

In the calcination process, the ceramsite was modified with the modifier FeCl_3_·6H_2_O concentration of 1 mol/L by calcination for 3 h at a temperature of 300, 400, 500, or 600 °C. The prepared modified ceramsite samples were used in a small biological trickling filter tower for microbial biofilm experiments. The dry weight of each biofilm was measured when the biofilm of biotrickling filter was stable.

**Figure 2:**
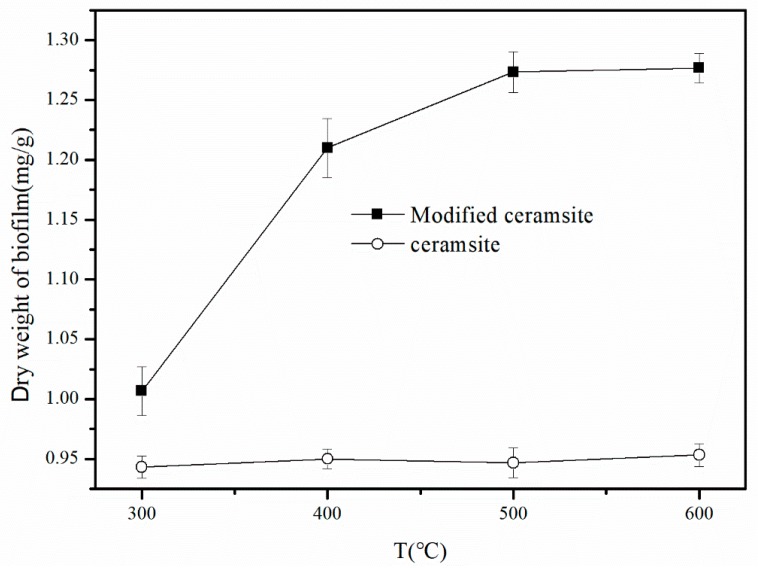
Dry weight of biofilm under different calcination temperature (300, 400, 500 and 600 °C) of the modified ceramsite.

As shown in Figure 2, the dry weight of the biofilm was 1.01 mg/g for the ceramsite modified at a calcination temperature of 300 °C and it increased by about 20% to reach 1.21 mg/g when the calcination temperature was 400 °C. The mass increased a bit more when the calcination temperature was raised up to 500 °C and then the mass remained constant when the calcination temperature was 600 °C. Based on these results, we selected 500 °C as the calcination temperature for ceramsite modification.

#### 3.1.2. Calcination Time

To determine the effect of calcination time on ceramsite modification, experiments were performed with a modifier concentration of 1 mol/L, calcination temperature of 500 °C, and calcination times of 1, 2, 3, 4, 5, and 6 h. The prepared modified ceramsite samples were then used in small biological trickling filter towers for microbial biofilm experiments. The dry weight of each biofilm was measured when the biofilm of each biotrickling filter was stable.

**Figure 3:**
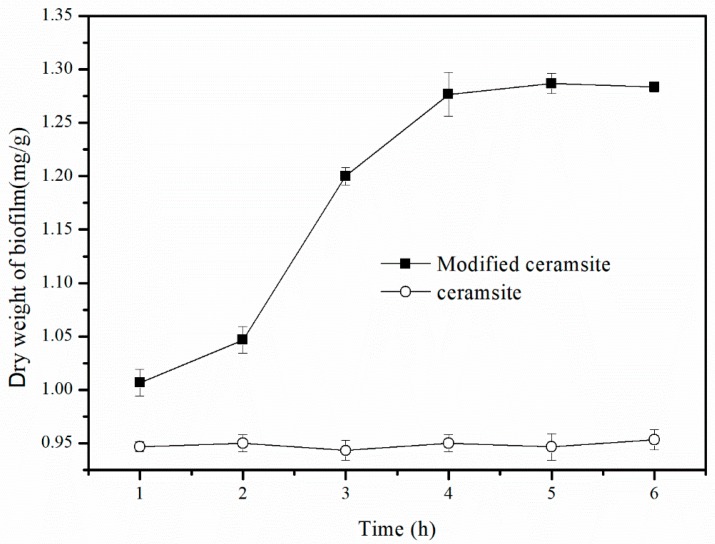
Dry weight of biofilm under different calcination time (1, 2, 3, 4, 5 and 6 h) of the modified ceramsite.

Figure 3 reveals that there was no obvious modification effect when the calcination time was 1 h, because the dry weight of biofilm was only 1.01 mg/g, which was only a slight increase over that of the case for unmodified ceramsite. The same phenomenon occurred when the calcination time was 2 h. The dry weight of the biofilm increased sharply, reaching 1.2 mg/g (an increase of nearly 20%) when the calcination time was extended to 3 h. When the calcination time was 4 h, the maximum dry weight of the biofilm of 1.28 mg/g was obtained and then stayed quite stable with the further lengthening of calcination time. According to the change of the dry weight of the biofilm, the crystal state of the surface was stable after calcination for 4 h, and the number of microbes adsorbed on the surface did not change when the calcination time was prolonged further. Therefore, we determined that the optimal calcination time was 4 h at 500 °C.

### 3.2. Properties of Modified Ceramics

**Figure 4:**
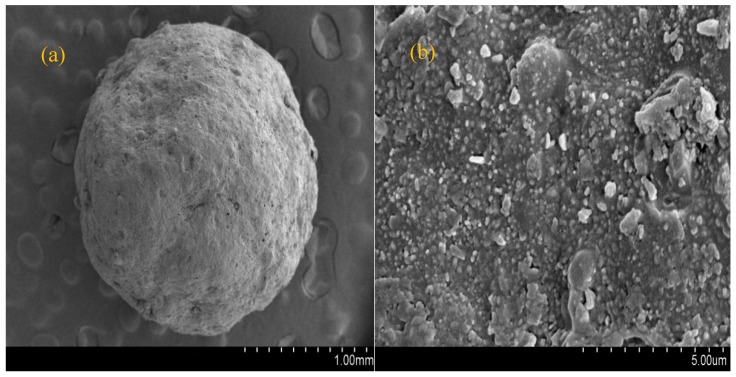
Scanning electron micrograph of commercial ceramsite: (**a**) 1 mm; (**b**) 5 µm.

**Figure 5:**
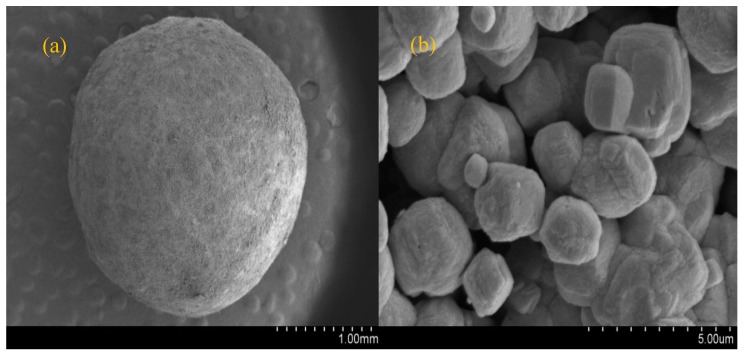
Scanning electron micrograph of modified ceramsite: (**a**) 1 mm; (**b**) 5 µm.

The purpose of modification is to change the surface properties of the ceramic which is reflected by the physical and chemical parameters, purification efficiency, and stability of the modified ceramic. The modification conditions were determined experimentally. The surface physical and chemical properties of the modified ceramsite were suitable to realize strong adsorption and immobilization of nitrogen removal bacteria. The physical and chemical properties of the ceramsite surface were changed by modification. Characteristic parameters of the unmodified and modified ceramsite samples are listed in Table 2. SEM images of the unmodified and modified ceramics are presented in Figure 4 and Figure 5, respectively.

Table 1 indicates that the shape and particle size of ceramsite did not change after modification, but its density increased by 17% and porosity increased by about 15%. In addition, modification increased the PI of ceramsite by more than four times and lowered the surface pH to 3.46. The surface pH is much lower than PI, which confirms that the surface is electropositive.

Figure 4 and Figure 5 illustrate that the modified ceramsite has a rougher surface compared with that of ceramsite; modification changed its two-dimensional rough surface into a three-dimensional surface. The modified ceramsite surface was thickly covered with crystals. The crystal morphology was trigonal and consistent with hematite, confirming the existence of a stable form of iron oxide on the modified ceramic surface. That is, the surface coating process successfully coated iron oxide on the ceramic surface. There are studies showing that crystal structure affects adsorption performance. For example, Bruno et al. [33] studied the sorption of phosphorus from aqueous solutions by crystalline and amorphous blast furnace slags. They found that the two slags had quite different sorption properties for phosphorus even though they had nearly the same compositions, which was attributed to their dissimilar morphological structures resulting in different surface areas. The surface area of crystalline slag was 0.65 m^2^/g, whereas that of amorphous slag was only 0.5 m^2^/g.

### 3.3. Start-up Performance of the Biotrickling Filter

**Figure 6:**
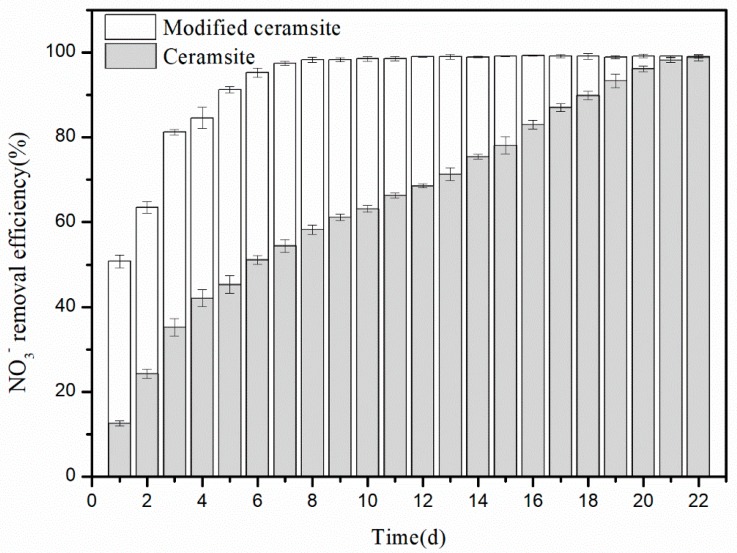
NO_3_^−^ removal efficiencies for modified ceramsite and commercial ceramsite as fillers in the biotrickling filter during the start-up period.

A previous study revealed that NO injection is not required until the denitrifying ratio reaches 80% and a macroscopic yellow biofilm appears [34]. Therefore, we replaced NO with KNO_3_ during the start-up period of the biotrickling filter. During the start-up period, the trickling rate was controlled at 250 mL/min and 50% of the trickling liquid was replaced every other day. To maximize cell adhesion to the packing medium, the reactor was operated in closed-loop mode. The NO_3_^−^ removal efficiency was considered the indicating factor in the start-up period. To obtain a reliable variation of the NO_3_^−^-N removal efficiency, a stable initial NO_3_^−^-N concentration for each day of the start-up period should be maintained, hence, the external nitrogen (KNO_3_) concentration of the newly added trickling liquid was variable due to the residual NO_3_^−^-N concentration and the initial NO_3_^−^-N concentration was ranged from 136 to 145 mg/L for each day during the whole startup phase. The results are presented in Figure 6. Generally, the formation of a biofilm in biological trickling filters is more difficult and requires a longer time compared with that in common biological filters [35]. This is mainly because the moving liquid has a shearing action on the packing; therefore, the adherent microbes must overcome this scouring force to grow. In addition, because microbial cells are covered in negative charges under natural conditions and commonly used packing material surfaces are also usually negatively charged, charge repulsion might hinder microbe adsorption on the filler. Thus, the start-up period of biotrickling filters is usually long (more than 20 days) with various type packing materials [36,37]. Some researchers [38] reported an enhanced system which featured a shorter start-up period with the inoculation of the enriched denitrifying bacteria significantly reducing its acclimation time to 17 days. As shown in Figure 6, when commercial ceramsite was used as a filler, the biofilm took about 22 days to fully mature. Conversely, it only took 8 days for the biotrickling filter to start up when the modified ceramsite was used as the packing material. That is, ceramsite modification greatly shortened the start-up period. These results indicated that the biofilter packed with the modified filler could be quickly started up by inoculation with *C. daeguensis* TAD1.

### 3.4. Performance of the Biotrickling Filter

**Figure 7:**
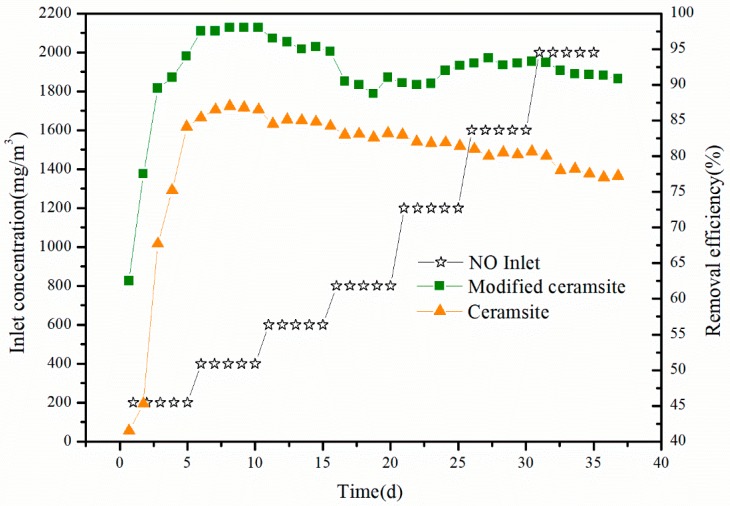
NO concentrations and removal efficiencies for modified ceramsite and natural ceramsite as fillers in the biotrickling filter during the operation period.

We used the modified ceramsite and commercial ceramsite as packing material in different biofilters and then tested the biofilter performance for NO removal under thermophilic conditions for 35 days after start-up. The bench-scale biofilter reactors (see Figure 1) were operated at high temperature (~50° C). The circulating fluid (pH = 7) was sprayed at a rate of 250 mL/min, and the empty bed residence time (EBRT) was 88 s. The inlet NO concentration was increased from 200 to 2000 mg/m^3^. The results are shown in Figure 7. For the biofilter with modified ceramsite, the removal efficiency was less than 80% during the first three days, which was because NO suddenly became the only nitrogen source when it replaced KNO_3_. However, the removal efficiency increased appreciably after 3 days. When the inlet NO concentration was increased from 200 to 2000 mg/m^3^ and EBRT was 88 s, the removal efficiency of NO did not apparently decrease and maintained an average value of above 90% during the remaining 32 days of treatment. The biofilter containing commercial ceramsite displayed lower removal efficiency compared with that of the biofilter with the modified ceramsite; in particular, at a high NO inlet concentration of above 1600 mg/m^3^, the removal efficiency was less than 80%. The biofilter with the modified filler exhibited stable and efficient NO removal performance compared with that of the unmodified case, and this effect was more prominent at high NO inlet concentration. The iron oxide-modified ceramsite improved the performance of the biofilter for NO removal.

The maximum inlet loading of the modified ceramsite biofilter was 80 g/(m^3^·h) with an average removal efficiency of 91.1%, which revealed that the maximum elimination capacity was much higher than that of some typical biofilters as shown in Table 3. In general, the capability of the modified ceramsite biofilter for NO removal was excellent and presented a promising practical application for NO removal with strong adaptability under various conditions especially for the high inlet loading.

### 3.5. Analysis of Surface Stability

**Figure 8:**
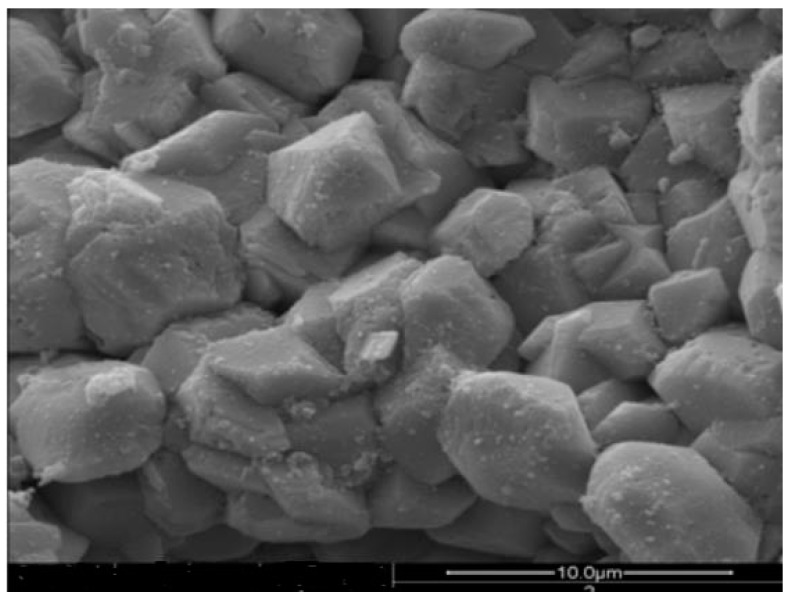
Scanning electron micrograph of modified ceramic after 2160-h use.

The performance of ceramsite as a filler was greatly improved following modification with an iron oxide-based coating on its surface. To investigate the stability of the ceramic surface coating, the modified ceramsite was packed in a biotrickling filter before start-up and operation over a continuous period of 90 days, and then removed for morphological observation (Figure 8). Compared with that of the unused modified filler surface (Figure 5), the crystal structure became clearer. This was attributed to the gradual loss of the amorphous component during operation, whereas the stable crystal structure was not removed so easily; therefore, the surface of the iron oxide-coated ceramsite remained intact. The loss of the surface coating was not obvious under the investigated experimental conditions, indicating that the coating is relatively stable when used in a biological filter system during long-term operation.

## 4. Conclusions

The performance of iron oxide-modified ceramsite as a filter medium in a biotrickling process was investigated. SEM results suggested that the modified ceramsite possessed a uniform interconnected crystal structure, which was more suited to microbial growth than the compact and closed pore structure of commercial ceramsite. It only took 8 days for the biotrickling filter with the modified ceramsite to start up, which was much shorter than the start-up period of the filter with commercial ceramsite. The filter packed with the modified filler displayed stable NO removal performance that was more efficient than that of the biofilter with the unmodified ceramsite; this effect was more prominent at high NO inlet concentration. The iron oxide-coated porous ceramsite thus improved the NO removal performance of the biofilter. The loss of the surface coating was not obvious under the experimental conditions and the coating remained relatively stable during operation for a long period. These observations reveal the promise of iron oxide-coated ceramsite as packing material in biotrickling filters for NO removal under thermophilic conditions.

## Figures and Tables

**Table 1:** The operating conditions for each packing material.

Packing Material	Start Up (d)	NO_3_^−^-N Concentration (mg/L)	pH	Operation (d)	NO Inlet (g/m^3^)	EBRT (s)	T (°C)
Ceramsite	22	136–145	7–7.5	35	0.2–2	88	50 ± 1
Modified ceramsite	8	136–145	7–7.5	35	0.2–2	88	50 ± 1

**Table 2:** Characteristic parameters of ceramsite and modified ceramsite.

	Shape	Diameter (mm)	Coating Contents (mg/g)	Density (g/m^3^)	Surface Area (m^2^/m^3^)	Porosity (%)	PI	Surface pH
Before	sphere	3–5	0	1.98	398	48	0.7–3	6.95
After	sphere	3–5	42.1	2.36	398	55	8.5	3.46

**Table 3:** Performance of some typical bioreactors for NO removal.

Filler	Temperature (°C)	O_2_ (%)	NO Inlet (mg/m^3^)	EBRT (min)	Inlet Loading (g/(m^3^·h))	RE (%)	Reference
modified PVC	50 ± 0.5	1–3	315	1	18.75	75	[39]
soil	20–37	-	335	-	-	60	[40]
ceramics	50 ± 0.5	2–20	800	1.8	26.67	80–92	[41]
ceramics	30 ± 0.5	2–20	800	1	48	63	[42]
woven fiber	50 ± 1	8	2000	0.7	163.6	89.8	[34]
modified ceramsite	50 ± 1	8	2000	1.5	80	91.1	This study
